# Effects of Oxytetracycline and Gentamicin Therapeutic Doses on Hematological, Biochemical and Hematopoietic Parameters in *Cyprinus carpio* Juveniles

**DOI:** 10.3390/ani10122278

**Published:** 2020-12-03

**Authors:** Elżbieta Kondera, Bartosz Bojarski, Katarzyna Ługowska, Barbara Kot, Małgorzata Witeska

**Affiliations:** 1 Institute of Biological Sciences, Faculty of Exact and Natural Sciences, Siedlce University of Natural Sciences and Humanities, Prusa 14 Street, 08-110 Siedlce, Poland; elzbieta.kondera@uph.edu.pl (E.K.); katarzyna.lugowska@uph.edu.pl (K.Ł.); barbara.kot@uph.edu.pl (B.K.); malgorzata.witeska@uph.edu.pl (M.W.); 2 Institute of Ichthyobiology and Aquaculture in Gołysz, Polish Academy of Sciences, Kalinowa 2, 43-520 Chybie, Poland

**Keywords:** antibiotics, erythrocytes, head kidney, hepatic aminotransferases, fish, leukocytes, toxicity

## Abstract

**Simple Summary:**

Hematological, biochemical and hematopoietic effects of therapeutic doses of oxytetracycline (OTC) and gentamicin (GEN) in clinically healthy common carp juveniles were studied. The fish were divided into four groups: controls 1 and 2 (untreated or injected with 0.6% NaCl solution), and two groups treated with antibiotics (orally with OTC four times every two days or injected with a single dose of GEN dissolved in 0.6% NaCl). Blood and head kidneys were sampled from all fish 3 days post-treatments for hematological, biochemical and hematopoietic tissue analyses. The obtained results showed no considerable hematotoxicity or hepatotoxicity of therapeutic doses of OTC and GEN to carp.

**Abstract:**

Hematological, biochemical and hematopoietic effects of therapeutic doses of two antibiotics, oxytetracycline (OTC) and gentamicin (GEN), in clinically healthy common carp juveniles were studied. The fish were divided into four groups: controls 1 and 2 (untreated or injected with 0.6% NaCl solution), and two groups treated with antibiotics (orally with 75 mg/kg OTC four times every two days or injected with a single dose (4 mg/kg) of GEN dissolved in 0.6% NaCl). Blood and head kidneys were sampled from all fish 3 days post-treatments for hematological, biochemical and hematopoietic tissue analyses. No major alterations in the values of hematological and serum biochemical parameters occurred following administration of OTC or GEN. Glucose concentrations were significantly lower in both groups of fish subjected to injections (Control 2 and GEN), while the oxidative metabolic activity of phagocytes increased in the antibiotic-treated groups (significantly in OTC). More alterations were observed in hematopoietic tissue. Immunocytochemical analysis revealed that G caused a significant increase in the rate of cell proliferation (PCNA-positive cells) and an increase in the frequency of apoptotic cells (caspase-positive). The frequency of lymphoid lineage decreased, which was related to a decrease in the abundance of mature lymphocytes in GEN-treated fish. Percentages of neutrophilic lineage were significantly elevated in OTC and GEN groups compared to controls. The obtained results showed no considerable hematotoxicity or hepatotoxicity of therapeutic doses of OTC and GEN to carp.

## 1. Introduction

Fish rearing under high stocking densities is accompanied by increased risk of infectious bacterial diseases that are treated mainly with antibiotics. Oxytetracycline (OTC) is often employed in fish farms to treat or prevent bacterial infections [1] and is one of the most used antibiotics in aquaculture [2]. Gentamicin (GEN) is an aminoglycoside antibiotic commonly used in veterinary medicine against a variety of infections caused by Gram-positive and Gram-negative aerobic bacteria and sometimes applied in fishery practice [3]. Aminoglycosides are also used in prophylaxis or as growth promoters for farm animals [4]. The presence of aminoglycosides in fresh water and sediments was reported in Polish rivers and lakes [5]. Irrational use of antibiotics accelerated the spread of antibiotic resistant bacteria across the globe [6]. Antibiotics at significant concentrations in aquatic environments are the principal source of selective pressure for antimicrobial resistance in bacteria because susceptible bacteria in the sediment and water are replaced by resistant ones [7], while antibiotics at subinhibitory concentrations may stimulate mutagenesis and horizontal transfer of antibiotic resistance genes (ARGs) for human pathogens, which may result in decreased efficacy of different antibiotic groups and severely limits the therapeutic options in human infections [8,9]. OTC is an antibiotic belonging to the tetracycline class. In fish farm environments where oxytetracycline was used, higher prevalence of tetracycline resistance (*tetR*) genes was showed than in those in which this antibiotic was not applied [10,11].

Moreover, OTC and GEN may show adverse effects on target organisms. Elia et al. [1] reported disturbances in the hepatic and renal antioxidant systems in *Cyprinus carpio* treated with 150 and 300 mg/kg of OTC, while the therapeutic dose (75 mg/kg) caused only minor changes—an increase in glutathione peroxidase and glutathione reductase activities. Hepatic and renal histopathological changes were observed by Reda et al. [12] in *Oreochromis niloticus* fed for 12 weeks diet containing 100 mg/kg of OTC. Histopathological alterations in the gills and liver of *Oncorhynchus mykiss* after acute exposure to OTC (96 h: 0.005–50 mg/L) were reported by Rodrigues et al. [13]. According to Grondel et al. [14], OTC delayed mitogenic response of phytohaemagglutinin-stimulated pronephric lymphocytes in *Cyprinus carpio*. Botelho et al. [15] reported a genotoxic effect of waterborne environmentally realistic OTC levels (4–16 µg/L) in *Oreochromis niloticus*. Mild oxidative effect and genotoxic damage was reported in OTC-treated *Oncorhynchus mykiss* by Rodrigues et al. [16]. It was confirmed by the results obtained by Pes et al. [17] who reported oxidative stress in *Rhamdia quelen* fed for 21 days with feed containing 0.1 g/kg of OTC. The fish showed increased lipid and protein peroxidation accompanied by an increase in activities of superoxide dismutase, glutathione peroxidase and glutathione reductase. Oxytetracycline caused delayed hatching of *Danio rerio* embryos, inhibited catalase and induced glutathione-S-transferase and lactate dehydrogenase activities in adults [18]. The larvae of *Danio rerio* exposed to 125–1500 mg/L of OTC showed a dose-dependent increase in neutrophil count, indicating an inflammatory response [19]. Various hematological effects of OTC were also reported but the results of various studies are contradictory. Omoregie and Oyebani [20] observed dose-dependent anemia, leukopenia and throbocytopenia in *Oreochromis niloticus* fed for 8 weeks with feed containing 0.63–5% OTC. Leukopenia with neutropenia were observed in *Oncorhynchus mykiss* fed 2.5 g/kg of OTC [21]. Neutropenia and monocytosis occurred in *Oncorhynchus mykiss* after injection of 20 mg/kg of OTC [22]. Increases in the values of hemoglobin concentration, hematocrit and leukocyte count accompanied by a decrease in erythrocyte count were reported by Ambili et al. [23] in *Labeo rohita* exposed to waterborne OTC (80 mg/L) for 25 days. No alterations in most hematological values except for a decrease in hematocrit and thrombocyte count were observed in *Oreochromis niloticus* fed a diet containing 500 mg/kg of OTC for 2 weeks [24]. No changes in the values of most hematological parameters and thrombocytosis in the same species fed a similar diet for 60 days were reported by El-Adawy et al. [25]. A short-term increase in erythrocyte and leukocyte counts in *Sparus aurata* after dietary administration of 75 mg/kg of OTC for 10 days was reported by Serezli et al. [26]. OTC-induced alterations in plasma biochemical profile included increases in the activities of hepatic alanine aminotransferese (ALT) and aspartate aminotransferase (AST) [21,25,27].

The main limitation of gentamicin use is its potential nephrotoxicity [28]. Reimschuessel et al. [29] reported progressive necrosis of proximal renal tubules of *Opsanus tau* in 2–8 days after intraperitoneal or intramuscular injection of 2.5–50 mg/kg of GEN. However, in *Carassius auratus*, regeneration of injured nephrons and entirely new nephrons were observed within 2–3 weeks after injection of 50 mg/kg of GEN [30]. *Oreochromis nilotica* showed acute tubular necrosis after intraperitoneal injection of 5 or 25 mg/kg of GEN followed by regeneration of epithelial cells of damaged tubules and the development of new nephrons [31]. According to Chen et al. [32], GEN (36 mg/kg) induced significant changes in the trunk kidney, liver, and intestine accompanied by anemia (reduced hematocrit and erythrocyte count) in *Oreochromis nilotica*. Gentamicin-treated fish also showed reduced levels of plasma sodium, chloride, calcium and iron, decreased total protein and cholesterol levels and hyperglycemia, accompanied by considerably elevated activities of alanine aminotransferase, aspartate aminotransferase, and creatine kinase. The data obtained by Cernaro et al. [28] revealed tubular degeneration with loss of the cellular apical brush border, necrosis and hyaline inclusions in the cytoplasm of renal tubule epithelial cells of *Danio rerio* injected with 0.4 mg of GEN. Exposure to 0.001% gentamicin sulfate solution for 24 h induced damage to the lateral line neuromasts of *Astyanax fasciatus* and *Danio rerio* [33].

The available data on hematological effects of oxytetracycline and gentamicin in fish are scarce and ambiguous, and nothing is known about the possible effects of these antibiotics on hematopoietic processes. Therefore, in the present study the issue of hematological and hematopoietic effects of therapeutic doses of these antibiotics used in healthy juvenile common carp was undertaken to broaden the knowledge of their possible adverse side effects.

## 2. Materials and Methods

Common carp *Cyprinus carpio* L. healthy juveniles of body mass 65.8 ± 7.6 g and body length 14.7 ± 6.1 cm were harvested from the rearing ponds of the Inland Fisheries Institute in Żabieniec, Poland in autumn and transferred in plastic bags with water and pure oxygen to the laboratory of the Department of Animal Physiology, Siedlce University of Natural Sciences and Humanities. The fish were stocked into the flow-through aerated 300 L tank supplied with fresh nonchlorinated tap water (initial temperature 12 °C and oxygen concentration 9.0 mg/L) and acclimated for 3 months during which the temperature was gradually raised to 17.5 °C, the oxygen concentration was 7.6–8.5 mg/L, the pH was 7.0–7.5, and ammonium and nitrite were 0.0 mg/L. During the acclimation period, the fish were fed once a day to satiation with pellets Aller Aqua Classic 4 mm diameter (containing 30% protein, 7% lipid, 43% carbohydrate, 7% ash, 5% fiber and with a calorific value of 433 kcal/100 g). Before the experiment, 40 fish were randomly transferred to 4 aerated glass aquaria of 100 L volume, 10 fish in each and left for a week to habituate. During this time, the fish were fed with Aller Aqua Classic 4 mm pellets (Aller Aqua, Golub-Dobrzyń, Poland) once a day in the morning at the rate of 1% of body mass. Every day, about 3 h post feeding, ¾ of water was gently siphoned out so as not to disturb fish and immediately replaced with fresh tap water to maintain appropriate water quality.

After the period of habituation, the fish from groups Control 1 and 2, and GEN, were fed as previously described. The fish from group OTC were fed every 2 days (4 times) with Aller Aqua Classic 4 mm pellets mixed with an oxytetracycline hydrochloride preparation dedicated to fish (Ichtioxan, Biofaktor, Skierniewice, Poland) at the dose of 75 mg/kg. OTC was administered according to the producer’s protocol. Ichtioxan contains 750 mg/g of powdered oxytetracycline hydrochloride, therefore to obtain recommended therapeutic dose of 75 mg/kg, 100 mg of Ichtioxan per 1 kg of fish mass was used. Briefly, a preweighed amount of pellets (1% of fish mass) was soaked in about 5 mL of aqueous solution containing 65.8 mg of Ichtioxan, gently mixed and dried at 50 °C. A similar method of OTC oral administration was described by Trushenski et al. [34], while the results obtained by Hassani et al. [35] showed that OTC is thermostable. On every second day, the fish from the OTC group obtained plain feed. On the day 8, the Control 2 fish were intraperitoneally injected with a single dose of sterile 0.6% NaCl solution, while GEN fish were intraperitoneally injected with a single dose (4 mg/kg) of gentamicin (Biowet, Puławy, Poland) dissolved in 0.6% NaCl solution. No therapeutic GEN dosage for cyprinid fish was found in the literature (however, in fishery practice single injections of 4–5 mg/kg are often used); therefore, the dose was established based on the reports by Jones et al. [36] and Bojarski et al. [3] who applied 3.5 mg/kg or 5 mg/kg of GEN to *Carassius auratus* and *Carassius gibelio*, respectively, in a single injection. Three days after the end of treatments (feeding OTC and injections) blood and head kidneys were sampled from all fish. A time of three days post treatments was left to develop reaction to antibiotics, particularly in the hematopoietic system.

Water quality parameters during habituation and the experimental period were measured every day (temperature, oxygen concentration) or every 3 days (pH, ammonium and nitrite concentrations). Temperature and oxygen level were measured using an oxygen meter (HI 9143, Hanna Instruments, Woonsocket, RI, USA), pH with a pH-meter (N5123, Elwro, Wrocław, Poland), and nitrogenous metabolites using colorimetric Visocolor kits (Visocolor Eco Ammonium 3 and Visocolor Eco Nitrite, Macherey Nagel, Düren, Germany). During the habituation and experimental periods, water temperature was 18.0–19.5 °C, O_2_ concentration 7.1–7.5 mg/L, pH 7.3–7.6, ammonium 0.05 mg/L and nitrite 0.0 mg/L.

Blood was sampled from live fish by heart puncture with heparinized needles to heparinized Eppendorf tubes (2 tubes per fish—500 µL of blood for plasma to evaluate hepatic enzyme activities, and 200 µL for remaining hematological and biochemical analyses). The following analyses were performed using fresh full blood: hematocrit (Ht), erythrocyte count (RBC), measurement of hemoglobin concentration (Hb), calculations of mean cell volume (MCV), mean corpuscular hemoglobin (MCH) and mean corpuscular hemoglobin concentration (MCHC), leukocyte count (WBC), measurement of oxidative metabolic activity of phagocytes (NBT), measurement of plasma glucose. Blood smears were also made to evaluate blood cell morphology. Activities of alanine aminotransferase (ALT) and aspartate aminotransferase (AST) were measured in plasma. Hematocrit was measured using the microhematocrit method after centrifugation of capillaries with blood. Erythrocyte and leukocyte counts were performed in Bürker hemocytometers (Paul Marienfeld GmbH & Co. KG, Lauda-Königshofen, Germany) in blood diluted 100 times with Hayem solution. Hemoglobin concentration was measured spectrophotometrically at 540 nm wavelength after the mixing of blood with Drabkin solution and the conversion of hemoglobin to cyanmethemoglobin. Hb values were calculated from the equation of the relationship between dilutions of hemoglobin standard and their extinction. MCV, MCH and MCHC were calculated using Ht, Hb and RBC values using standard formulas [37]. Glucose concentration was measured using an Accu Check (Roche, Basel, Switzerland) glucometer. Oxidative metabolic activity of phagocytes (NBT) was measured spectrophotometrically at 546 nm wavelength using the nitrotetrazolium blue reduction method adopted for fish blood [38]. ALT and AST activities were measured spectrophotometrically at 340 nm wavelength in blood plasma obtained by blood centrifugation and using Pointe Scientific kits (Pointe Scientific, Warsaw, Poland), according to the producer’s protocol. Blood smears were stained using May–Grünwald and Giemsa solutions and fixed with Histokitt (Carl Roth, Karlsruhe, Germany) and cover slips. They were inspected using a Nikon Eclipse E600 light microscope (Nikon, Tokyo, Japan) at 400× magnification. In each smear, 300 erythrocytes, 100 leukocytes and the number of thrombocytes accompanying the 100 leukocytes were counted. Percentage of erythroblasts and abnormal erythrocytes, differential leukocyte count (percentage of lymphocytes, neutrophils and monocytes), thrombocyte count (TC) based on the number of thrombocytes in smear per 100 leukocytes and WBC values were calculated.

Then, all fish were euthanized with MS-222 (Sigma-Aldrich, Poznań, Poland) overdose and decapitated. Head kidneys were isolated and hematopoietic tissue preparations were made. The surface of isolated fresh organs was blotted, and then tissue was gently smeared on degreased slides. After being dried at room temperature for 24 h, the smears were stained using May–Grünwald and Giemsa solutions. Then, the smears were viewed using a light microscope (Nikon Eclipse microscope 300, at 1000× magnification, Nikon Corporation, Tokyo, Japan) to evaluate cellular structure of hematopoietic tissue. Percentages of all types of hematopoietic precursor cells (22 types of cells) were calculated per 500 cells inspected in each smear, according to Fijan [39,40] and Kondera [41]. The identified cells were then grouped into the main cell lineages (erythroid, lymphoid, neutrophilic, monocytoid, basophilic, eosinophilic, thrombocytoid), most of which included various developmental stages. Early blast cells of various lineages were morphologically similar, so they were included in one common group to avoid identification bias. Cells of unclear characteristics were placed into the group of unclassified cells. Fields with crowded, deformed, damaged cells were excluded. Areas of kidney tissue with numerous mature erythrocytes (possibly blood vessel contents) were also ignored.

The other smears were stained using immunocytochemical methods to visualize PCNA-positive cells (indicating proliferative activity) and caspase 3-positive cells (indicating apoptotic activity). The presence of PCNA was detected using rabbit monoclonal anti-PCNA antibodies (Dako, Carpinteria, CA, USA) and visualized using the Dako rabbit PCNA Envision system. The presence of caspase 3 was detected with mouse anti-caspase 3 active antibodies (Sigma-Aldrich, Poznań, Poland), and visualized using the Dako mouse anti-caspase Envision system. The reagents were used according to the producer’s protocols. The PCNA-positive and caspase 3-positive cells stained brownish and were easily distinguishable from other cells (stained light blue with hematoxylin). Percentages of proliferating (PCNA-positive) and apoptotic (caspase 3-positive) precursor cells were counted in at least 6 fields per 300 inspected hematopoietic cells, and the hematopoietic cell turnover rate was calculated as the PCNA/caspase 3 ratio.

The results were subjected to statistical analysis using Statistica 12 (Dell Technologies, Round Rock TX, USA). Mean values and standard deviations were calculated for the values of all parameters in the experimental groups and the Shapiro–Wilk test was used to evaluate the normality of distribution. As most parameters showed normal distribution, one-way ANOVA followed by Duncan’s post-hoc test were performed to evaluate significance of differences in the values of parameters among the groups (assuming significance level *p* ≤ 0.05).

The study was performed according to the Act on the Protection of Animals Used for Scientific and Educational Purposes and with the consent of the Local Ethics Committee in Warsaw (No—767/2018).

## 3. Results

No major alterations in the values of hematological parameters occurred following administration of OTC or GEN and no significant differences were observed among the experimental groups (Table 1). Antibiotics did not significantly alter the activities of hepatic aminotransferases ALT and AST; however, in groups exposed to antibiotics the results were much more variable and the mean values were higher compared to the controls. The only significant differences were observed in the values of plasma glucose level and NBT. Glucose concentrations were significantly lower in both groups of fish subjected to injections (Control 2 and GEN), while NBT values were elevated in antibiotic-treated groups (significantly in OTC).

**Figure 1:**
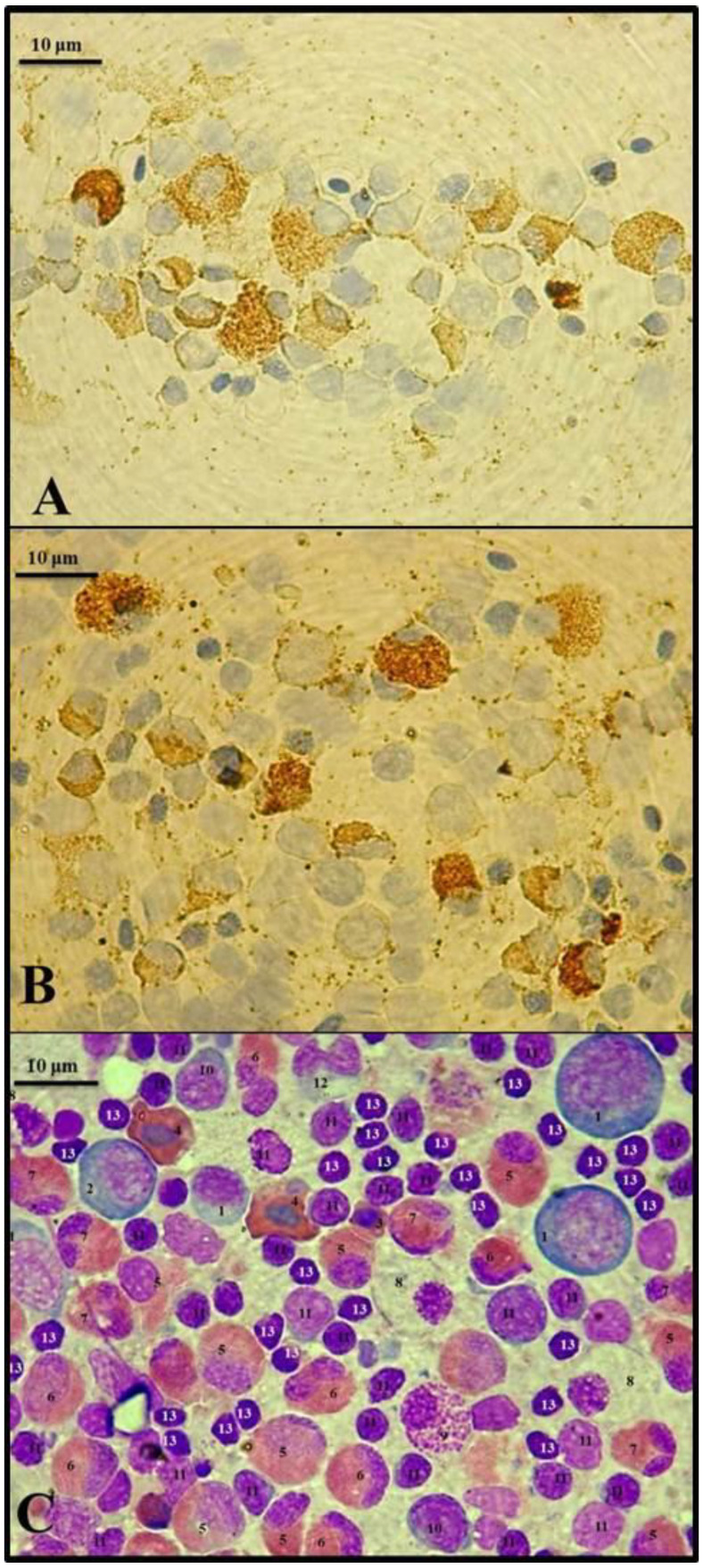
Blood cells in *Cyprinus carpio* head kidney: (**A**)—PCNA-positive cells, (**B**)—Caspase 3-positive cells (stained brownish, immunocytochemical staining), (**C**)—May–Grünwald and Giemsa solutions (1—blast cell; 2—basophilic erythroblast; 3—young erythrocyte; 4—mature erythrocyte; 5—myelocyte; 6—metamyelocyte; 7—segmented neutrophil; 8—proglanulocyte; 9—mature basophil; 10—prolymphocyte; 11—lymphocyte; 12—promonocyte; 13—thrombocyte).

Antibiotic treatments resulted also in some significant changes in the head kidney hematopoietic tissue of carp (Table 2). Immunocytochemical analysis revealed that GEN caused a significant increase in the rate of cell proliferation (PCNA-positive cells) (Figure 1A) compared to Control 2, and an increase in the frequency of apoptotic cells (caspase 3-positive) (Figure 1B) compared to both Control 1 and 2. Mean frequencies of proliferating and apoptotic cells in the OTC group were higher compared to the Controls, but the differences were statistically insignificant. No significant differences occurred in the cell turnover rate measured as the ratio of proliferating to apoptotic cells (PCNA/caspase 3). In addition, no significant differences occurred in the percentages of most cell lineages, among which only the frequency of lymphoid and neutrophilic cells significantly changed. The frequency of lymphoid lineage in GEN group was significantly lower compared to the Control 1, which was related to a decrease in the abundance of mature lymphocytes. Percentages of neutrophilic lineage were significantly elevated in both OTC and GEN groups which resulted mainly from an increase in the abundance of immature neutrophils (myelocytes and metamyelocytes) (Figure 1C). The frequency of mature (band and segmented) neutrophils significantly increased only in the GEN group compared to the related Control 2.

## 4. Discussion

Antibiotics are commonly used to control bacterial diseases in both human and veterinary medicine. However, the main source of environmental load is currently intensive livestock production in which antibiotics are used for nontherapeutic purposes (prevention and growth promotion) [42]. Antibiotics used in animals, including oxytetracycline and gentamicin, are not completely absorbed or metabolized in the organisms to which they have been administered, and about 30–90% of the used amount is excreted through urine and feces into the environment [43]. The livestock manure is an important source of environmental contamination with antibiotics [44]. Antibiotics from manures can enter the soil, followed by ground and surface water. The presence of antibiotics in the aquatic environment is worrying because of the possibility of the emergence of resistant bacterial strains including resistant human bacterial pathogens [45], but also in the context of possible toxic effects on aquatic organisms, including fish.

The results of our study showed no significant alterations in peripheral blood parameters of common carp juveniles treated with therapeutic doses of oxytetracycline and gentamicin, except for an increase in the oxidative metabolic activity of phagocytes (NBT) which indicates possible alteration in nonspecific immune response. The data concerning hematological and immune response in fish after antibiotic treatment are scarce and ambiguous. The results obtained by various authors probably depend on antibiotic dosage and the sensitivity of various fish species. A similar effect to that recorded in the present study was reported by Serezli et al. [26], who observed an increase in the frequency of NBT-positive cells in *Sparus aurata* after oral OTC administration (10 days, 75 mg/kg). It was accompanied by a short-term increase in RBC and WBC. On the other hand, Reda et al. [12] reported a slight decrease in the phagocytic index in OTC-treated *Oreochromis niloticus*, while Maklakova et al. [22] found significant neutropenia and monocytosis in *Oncorhynchus mykiss* injected with five doses of 20 mg/kg of OTC. *Labeo rohita* subjected for 25 days to 80 mg/L of waterborne OTC showed an increase in Hb and Ht accompanied by a decrease in RBC, and an increase in WBC [23]. Omoregie and Oyebanji [20] observed distinct anemia, a significant decrease in RBC, Ht and Hb, as well as leukopenia and thrombocytopenia in the same species of fish fed diets containing 0.6–5% OTC for 8 weeks. Very little data are available on hematological effects of gentamicin in fish. Chen et al. [32] reported anemic response (decrease in Ht and RBC) in *Oreochromis niloticus* injected with 36 mg/kg of GEN after 18–20 h.

The results obtained in the present study revealed no significant alterations in AST and ALT activities in fish treated with OTC or GEN, which indicates no hepatotoxic effects of treatments. However, other authors reported that these antibiotics may induce hepatotoxicity. Excessive doses of dietary OTC (100 mg/kg body weight/day orally for 2 weeks) induced an increase in hepatosomatic index (liver enlargement) accompanied by the increase in ALT and AST activities in *Oncorhynchus kisutch* [27]. Similar results—specifically, increases in the activities of hepatic ALT and AST—were reported by Ambili et al. [23] for *Labeo rohita*. Reda et al. [12] observed a significant decrease in ALT and increase in AST activity in *Oreochromis niloticus* fed for 12 weeks with a diet containning 100 mg/kg of OTC. According to Chen et al. [32], GEN (36 mg/kg) increased activities of ALT and AST.

The results obtained in the present study showed that heamtopoietic tissue was more sensitive to antibiotics compared to the peripheral blood (more significant alterations were observed). The changes observed in antibiotic-treated groups confirm the effect found in peripheral blood (activation of phagocyte response) since the shift from lymphocytes to neutrophils occurred, particularly in the GEN group. This was accompanied by an increased ratio of both proliferative and apoptotic activity of hematopoietic precursors and might have been related to possible GEN-induced tissue lesions. No data are available in the literature concerning the effects of OTC or GEN on hematopoietic tissue composition or activity in fish. The data concerning the effects of various toxic substances on fish hematopoietic tissue showed that cadmium caused a significant increase in the apoptotic rate of hematopoietic precursor cells with only minor increase in the rate of cell proliferation, which resulted in a reduced hematopoietic potential. Short-term exposure to 6.5 mg/L of cadmium resulted in an increase in percentage of monocytoid cells and eosinophils in the head kidney, while long-term exposure to 0.65 mg/L induced a significant increase in the frequency of erythroid precursors and thrombocytes in the head kidney [46]. Analysis of head kidney hematopoietic tissue revealed that roundup at concentrations of 0.5 and 5.0 mg/L caused a significant increase in the rate of cell proliferation accompanied by an increase in the frequency of early blast cells. The frequency of monocytoid, eosinophilic, and basophilic lineage cells significantly increased in the herbicide-exposed fish compared to the control [47].

Treating fish with OTC may cause also other pathological alterations, including oxidative stress. Nakano et al. [27] exposed *Oncorhynchus kisutch* to OTC at the dose of 100 mg/kg/day for two weeks. They reported that total glutathione content in the liver, muscle and stomach of OTC-treated fish was higher compared to the control. Plasma total glutathione level in OTC-fed fish was also elevated. Rodrigues et al. [16] reported that OTC-exposed *Oncorhynchus mykiss* exhibited a mild pattern of antioxidant response, with modifications in catalase and glutathione peroxidase activities in the gills, and lipid peroxidation in the liver. Exposure to OTC may lead to the development of histological changes in various fish organs. Rodrigues et al. [13] revealed histopathological changes in *Oncorhynchus mykiss* after acute (0.005 to 50 mg/L; 96 h) and chronic (0.3125 to 5 μg/L; 28 days) OTC exposures. They reported progressive disorders in gills after acute exposure and regressive changes after chronic exposure. In the liver, circulatory (e.g., hemorrhage), regressive (e.g., pyknotic nucleus) and progressive (e.g., hypertrophy of hepatocytes) changes were noted, but only after acute exposure. After chronic exposure, inflammatory changes were observed. Rodrigues et al. [48] demonstrated tissue alterations in the gills and liver of *Sparus aurata* individuals acutely (96 h) and chronically (28 days) exposed to oxytetracycline (0.0004–400 μg/L). Some circulatory, regressive, progressive, and inflammatory alterations were noted in both tested fish organs in all exposed individuals. On the other hand, Markling et al. [49] showed that even high doses of orally administered OTC did not cause toxic effects in *Salvelinus namaycush*. In addition, GEN may result in the development of pathological disorders, although the available literature data focus mainly on nephrotoxic effects. Augusto et al. [31] studied the renal response to gentamicin (5 mg/kg and 25 mg/kg of body weight) in *Oreochromis nilotica*. Gentamicin exposure induced acute tubular necrosis that peaked in severity at 2 days following intraperitoneal injection of the higher dose and at 4 to 7 days following injection of the lower tested dose. Necrosis after exposure to the higher tested dose was more severe. The authors stated that regeneration of epithelial cells along the basement membrane of damaged tubules and the development of new nephrons were documented. Reimschuessel et al. [29] observed an extensive necrosis in the proximal tubes in *Opsanus tau* injected with GEN. The results obtained by Hentschel et al. [50] showed a decline in the glomerular filtration rate after exposure of *Danio rerio* larvae to GEN. On the other hand, Bojarski et al. [3] observed no changes in the reduced glutathione concentration and activity of superoxide dismutase, glutathione peroxidase and catalase measured in GEN-treated *Carassius gibelio* kidney (5 mg/kg). Moreover, no histological lesions in the kidney were noted.

Our results revealed that therapeutic doses of oxytetracycline and gentamicin caused no significant symptoms of hemato- or hepatotoxicity. However, increased oxidative metabolism of phagocytes, higher hematopoietic precursor cell proliferation and apoptotic rates and higher proportion of neutrophils in hematopoietic tissue of fish treated with antibiotics (particularly gentamicin) compared to the control groups indicate that treatments might have induced slight inflammatory lesions. 

## Figures and Tables

**Table 1:** The effects of oxytetracycline and gentamicin on the hematological and biochemical parameters in common carp (Control 1—untreated, Control 2—injected with 0.6% NaCl, OTC—fed oxytetracycline 4 times every 2 days at 75 mg/kg, G—injected with single dose 50 mg/kg gentamicin in 0.6% NaCl, different letter superscripts indicate statistically significant differences among groups, *n* = 10, Duncan post-hoc test, *p* ≤ 0.05).

Parameter	Experimental Groups			
	Control 1	Control 2	OTC	G
Ht [%]	30.7 ± 3.4 ^a^	33.4 ± 3.2 ^a^	32.7 ± 3.8 ^a^	30.9 ± 4.0 ^a^
Hb [g/L]	84.8 ± 13.0 ^a^	98.1 ± 13.2 ^a^	89.4 ± 10.4 ^a^	93.5 ± 7.5 ^a^
RBC [10^6^/µL]	1.60 ± 0.14 ^a^	1.71 ± 0.31 ^a^	1.74 ± 0.24 ^a^	1.65 ± 0.13 ^a^
MCV [fL]	193.0 ± 28.9 ^a^	199.0 ± 29.6 ^a^	190.6 ± 25.2 ^a^	188.4 ± 25.8 ^a^
MCH [pg]	53.4 ± 9.8 ^a^	58.5 ± 9.6 ^a^	52.2 ± 7.8 ^a^	57.1 ± 6.2 ^a^
MCHC [g/L]	276.0 ± 19.6 ^a^	293.7 ± 29.2 ^a^	274.5 ± 23.9 ^a^	306.6 ± 43.9 ^a^
Erythroblasts [%]	0.1 ± 0.2 ^a^	0.2 ± 0.2 ^a^	0.2 ± 0.2 ^a^	0.1 ± 0.2 ^a^
Abnormal erythrocytes [%]	3.2 ± 2.4 ^a^	2.9 ± 2.4 ^a^	2.4 ± 2.2 ^a^	2.7 ± 2.2 ^a^
WBC [10^3^/µL]	77.0 ± 25.7 ^a^	57.9 ± 21.0 ^a^	69.9 ± 19.7 ^a^	62.9 ± 21.4 ^a^
Lymphocytes [%]	96.0 ± 5.8 ^a^	97.0 ± 3.6 ^a^	95.8 ± 3.9 ^a^	93.7 ± 5.0 ^a^
Neutrophils [%]	1.6 ± 2.1 ^a^	2.0 ± 3.3 ^a^	2.5 ± 2.6 ^a^	4.9 ± 6.0 ^a^
Monocytes [%]	1.8 ± 4.0 ^a^	0.7 ± 0.8 ^a^	0.9 ± 1.0 ^a^	1.1 ± 1.4 ^a^
TC [10^3^/µL]	5.4 ± 7.7 ^a^	4.2 ± 5.6 ^a^	4.7 ± 6.0 ^a^	5.4 ± 6.3 ^a^
Glucose [mg/dL]	65.3 ± 7.2 ^a^	54.7 ± 8.0 ^b^	70.2 ± 8.5 ^a^	55.1 ± 12.4 ^b^
NBT [g/L]	0.69 ± 0.21 ^a^	0.77 ± 0.17 ^a^	1.49 ± 0.18 ^b^	1.03 ± 0.27 ^ab^
ALT [U/L]	74.0 ± 13.8 ^a^	57.5 ± 14.9 ^a^	81.1 ± 38.7 ^a^	70.0 ± 31.8 ^a^
AST [U/L]	67.0 ± 8.9 ^a^	65.4 ± 27.4 ^a^	86.0 ± 51.1 ^a^	91.1 ± 68.0 ^a^

**Table 2:** The effects of oxytetracycline and gentamicin on the proliferative and apoptotic activity and cellular composition of hematopoietic tissue in common carp (Control 1—untreated, Control 2—injected with 0.6% NaCl, OTC—fed oxytetracycline 4 times every 2 days at 75 mg/kg, G—injected with single dose 50 mg/kg gentamicin in 0.6% NaCl, different letter superscripts indicate statistically significant differences among groups, *n* = 10, Duncan post-hoc test, *p* ≤ 0.05).

Cell Types	Experimental Groups			
	Control 1	Control 2	OTC	G
PCNA-positive [%]	7.6 ± 1.3 ^ab^	7.4 ± 1.2 ^a^	8.5 ± 1.7 ^ab^	9.5 ± 1.9 ^b^
Cas-positive [%]	6.9 ± 1.1 ^a^	6.5 ± 1.3 ^a^	8.1 ± 1.7 ^ab^	8.8 ± 1.5 ^b^
PCNA/Cas	1.2 ± 0.4 ^a^	1.2 ± 0.3 ^a^	1.1 ± 0.4 ^a^	1.1 ± 0.2 ^a^
Early blast cells	8.9 ± 2.8 ^a^	8.9 ± 2.6 ^a^	9.9 ± 2.8 ^a^	10.7 ± 3.2 ^a^
Erythroid [%]	6.2 ± 1.6 ^a^	6.4 ± 1.6 ^a^	7.5 ± 1.7 ^a^	6.8 ± 2.7 ^a^
Erythroblasts	2.6 ± 1.1 ^a^	3.0 ± 1.2 ^a^	3.8 ± 1.0 ^a^	3.4 ± 2.0 ^a^
Erythrocytes	3.5 ± 1.1 ^a^	3.5 ± 0.9 ^a^	3.8 ± 1.0 ^a^	3.4 ± 1.2 ^a^
Lymphoid [%]	51.0 ± 5.3 ^a^	48.7 ± 4.7 ^ab^	46.3 ± 8.1 ^ab^	41.0 ± 7.3 ^b^
Prolymphocytes	2.5 ± 1.8 ^a^	2.7 ± 1.2 ^a^	2.3 ± 1.0 ^a^	3.0 ± 2.4 ^a^
Lymphocytes	47.2 ± 5.8 ^a^	44.6 ± 4.7 ^ab^	42.3 ± 8.5 ^ab^	37.5 ± 7.1 ^b^
Plasmocytes	1.3 ± 0.6 ^a^	1.4 ± 0.6 ^a^	1.8 ± 0.6 ^a^	1.6 ± 0.8 ^a^
Neutrophilic [%]	20.1 ± 1.5 ^a^	20.6 ± 1.2 ^a^	23.7 ± 1.6 ^b^	26.1 ± 1.8 ^b^
Myelocytes + metamyelocytes	15.4 ± 1.8 ^a^	17.9 ± 1.5 ^ab^	18.7 ± 2.5 ^b^	20.5 ± 3.0 ^b^
Band + segmented neutrophils	4.5 ± 1.4 ^ab^	2.7 ± 1.4 ^a^	4.9 ± 1.9 ^ab^	5.6 ± 2.5 ^b^
Monocytoid [%]	2.3 ± 1.5 ^a^	1.8 ± 1.1 ^a^	1.4 ± 0.6 ^a^	1.2 ± 0.5 ^a^
Promonocytes	1.8 ± 1.1 ^a^	1.3 ± 0.8 ^a^	1.0 ± 0.4 ^a^	0.8 ± 0.4 ^a^
Monocytes	0.5 ± 0.5 ^a^	0.5 ± 0.5 ^a^	0.4 ± 0.4 ^a^	0.4 ± 0.2 ^a^
Basophilic [%]	4.5 ± 1.5 ^a^	5.3 ± 1.9 ^a^	4.4 ± 2.7 ^a^	5.1 ± 2.7 ^a^
Proglanulocytes + metagranulocytes	2.3 ± 1.6 ^a^	2.6 ± 1.1 ^a^	2.4 ± 1.7 ^a^	3.5 ± 2.5 ^a^
Basophils	1.9 ± 0.7 ^a^	2.7 ± 1.6 ^a^	2.0 ± 0.6 ^a^	1.6 ± 0.9 ^a^
Eosinophilic [%]	0.1 ± 0.3 ^a^	0.2 ± 0.3 ^a^	0.2 ± 0.2 ^a^	0.3 ± 0.3 ^a^
Thrombocytoid [%]	6.2 ± 1.7 ^a^	5.9 ± 2.0 ^a^	6.0 ± 2.0 ^a^	6.3 ± 1.9 ^a^
Unclassified cells	1.3 ± 0.6 ^a^	1.4 ± 0.4 ^a^	1.5 ± 0.6 ^a^	1.6 ± 0.4 ^a^
